# The effect of a multimodal comprehensive care methodology for family caregivers of people with dementia

**DOI:** 10.1186/s12877-021-02373-w

**Published:** 2021-07-22

**Authors:** Masaki Kobayashi, Miwako Honda

**Affiliations:** Department of Geriatric Medicine, National Hospital Organization Tokyo Medical Center, 2-5-1, Higashigaoka, Meguro-ku, Tokyo, 152-8902 Japan

**Keywords:** Multimodal comprehensive care methodology, Caregiver burden, Dementia, Behavioural and psychological symptoms of dementia, Zarit burden interview, Behavioral pathology in Alzheimer’s disease

## Abstract

**Background:**

Caregivers experience social, physical and psychological burdens in caring for people with dementia. A study was conducted to assess the efficacy of a multimodal comprehensive care methodology training programme for the family caregivers of people with dementia.

**Methods:**

This research was an intervention trial with a quasi-experimental design. A total of 148 family caregivers of people with dementia participated in a multimodal comprehensive care methodology training programme for 6 hours (three times for 2 hours) in 3 months, which was followed by weekly delivery of information via postcard. The care burden of the caregivers was evaluated by the Japanese short version of the Zarit Burden Interview (J-ZBI) before the training, 1 month post-training and 3 months post-training (primary outcome). Each caregiver assessed the symptoms of the people with dementia for whom they provided care with the Behavioral Pathology in Alzheimer’s Disease (Behave-AD) (secondary outcome).

**Results:**

A total of 117 family caregivers (79%) were assessed 3 months after training. Over the course of the programme, the care burden significantly decreased from pre-training to 3 months post-training (*P* < 0.001). The mean care burden scores before, 1 month after, and 3 months after the intervention were 13.3, 10.9 and 10.6, respectively. The mean Behave-AD score of 101 people with dementia (68%) 3 months post-training was lower than that at pre-training, but the difference was not statistically significant (from 13.6 to 11.8, *P* = 0.005).

**Conclusions:**

The multimodal comprehensive care methodology training was associated with a reduction in the care burden of family caregivers. These findings suggest that randomized controlled trials with larger sample sizes are needed.

**Trial registration:**

UMIN Clinical Trials Registry (UMIN-CTR), UMIN000043245.

Registered 4 February 2021 – Retrospectively registered

## Background

Dementia is a major health problem that causes physical and mental burdens on patients and caregivers. Informal caregivers, primarily spouses and children, play a central role in the care and health preservation of people with dementia who live at home. Although cognitive deficits are the clinical indication of dementia, behavioural and psychological symptoms of dementia (BPSDs) are almost ubiquitous and can dominate disease presentation [1].

Managing BPSDs is one of the most challenging aspects of care, causing caregiver burden and upset [2]. Caregivers of people with BPSDs are more distressed and depressed than those who do not manage such behaviours [3]. There is emerging evidence that caregiver distress associated with BPSDs is a more important predictor of institutionalization and inpatient and emergency department use than the frequency and severity of BPSDs themselves [4–6]. Unfortunately, no effective treatment options for BPSDs are currently available to family caregivers. Typically, if a caregiver expresses concern about a BPSD to a physician, a sleep medication or anti-psychiatric medication is prescribed to control the symptom. However, medication is an ineffective and potentially dangerous strategy [7, 8]. On the other hand, non-pharmacologic strategies are recommended by multiple medical organisations and expert groups. Currently, some non-pharmacological approaches appear to be effective as interventions for family caregivers [9–12]. However, such approaches, which require intensive and time-consuming training, have not been translated enough to real-world care [9–12].

A previous systematic review by Feast et al. [13] revealed two main reasons for behaviours being reported as challenging by family caregivers who have difficulty dealing with BPSDs: changes in communication and relationships between people with dementia and family caregivers, resulting in feeling bereft, and perceptions of transgressions against social norms associated with misunderstandings about the behaviour of people with dementia. The authors noted that carers who retained the conceptualisation of their relative with dementia as the person they had always known and loved would be able to continue to have a fulfilling relationship with the person with dementia and that the companionship and feelings of care gained from the relationship would help reduce the caregiver’s perception of BPSDs as challenging, thus improving their ability to cope.

The French care methodology of Gineste-Marescotti, called Humanitude™, was performed extensively in several settings, including nursing homes and hospitals, in the last 40 years [14, 15]. Gineste and Marescotti developed Humanitude™ in 1979. The methodology is a multimodal comprehensive communication technique that uses on a humanist philosophy that highlights respect for individual liberty, autonomy and dignity. Humanitude™ refers to the set of particularities that allow us to feel that we are members of the human species and recognize other human beings as members of the same species. The methodology focuses on 4 elements of communication with patients: gaze, talk, touch, and assistance with standing up. All care is provided in a sequence consisting of 5 stages: 1) Notification (*Pre-preliminaries*), 2) Preparation (*Preliminaries*), 3) Integration of communication (*Sensory circle*), 4) Emotional consolidation (*Emotional consolidation*), and 5) Next appointment (*Appointment*). The goal of Notification (Stage 1) is to announce the presence of the caregiver, avoid surprise approaches and respect the patient’s privacy and autonomy. Preparation (Stage 2) represents the initial establishment of a relationship through the relationship pillars (gaze, speech and touch), and it allows the caregiver to obtain consent for the relationship from the person receiving the care. Integration of communication (Stage 3) includes the provision of care with a consistent positive emotional environment between the caregiver and the patient. At the end of the care, Emotional consolidation (Stage 4) is a stage of cognitive and mental stimulation that leaves a positive impression of the relationship and care in the emotional memory of the person receiving it, which facilitates consent to the relationship and the acceptance of future care. Next appointment (Stage 5) is the final moment of the relationship, in which commitment to future care is affirmed because the emotional memory is functioning even if patient has advanced dementia. Goodbyes are said during this stage, and a new meeting is scheduled, which prevents a feeling of abandonment [14].

The multimodal comprehensive care methodology training programme includes skills that may be used at home. The programme includes lectures, demonstrations and role-playing workshops to teach participants to use this methodology for the care of people with dementia at home. After each training session, participants received weekly postcards with tips of daily care based on the methodology for 3 months. The 12 postcards included tips with illustrations that consisted of 2 for sequence of care, 6 for general communication skills and 4 for communication skills depending on pathophysiology of dementia.

The present study determined whether the multimodal comprehensive care methodology training programme would reduce family caregiver burden in caring for people with dementia. We also investigated BPSDs in people with dementia.

## Methods

### Study design, setting, and participants

An intervention trial with a quasi-experimental design was performed. This study was designed as one of the geriatric friendly city projects in Fukuoka city. Participants were consecutively recruited via a public relations magazine of Fukuoka city, which is the 5th largest city of Japan, in November 2016. Participants were non-randomly sampled. Inclusion criteria included: 1) primary family caregiver for a patient who was clinically diagnosed with dementia; 2) residing with the homebound person with dementia; 3) caring for a person with dementia who was over 65 years of age; and 4) having no experience with multimodal comprehensive care methodology training prior to this research. The family caregivers and people with dementia were mailed an information leaflet and informed consent, which they were asked to fill out and return. If people with dementia were unable to provide informed consent, proxy consent from the family caregiver was obtained. A total of 148 participants were recruited. A monthly 2-h training programme was held three times in a conference room of ACROS Fukuoka, which is a public complex in Fukuoka city, from December 2016 to Feb 2017.

### Procedure

Participants who consented to the study were sent pre-training survey forms as the baseline assessment. Participants completed the Japanese short version of the Zarit Burden Interview (J-ZBI) to assess caregiver burden from a few weeks before the training to the first day of the training [16, 17]. They also completed the Behavioral Pathology in Alzheimer’s Disease (Behave-AD) to evaluate behavioural and psychological symptoms in the people with dementia for whom they provided care [18, 19]. Participants provided background information of family caregivers, including the caregiver’s age, gender and relationship with the person with dementia, and the background information of the person with dementia, including age, gender, aetiology of dementia, support or care need levels under the public long-term care insurance system in Japan, and the use of any sleep or antipsychotic medications.

A monthly 2-h multimodal comprehensive care methodology training programme was provided 3 times. The training programme was delivered to approximately 20 family caregivers at a time. Three certified instructors, i.e., two nurses and a physiotherapist, who had 10 weeks of training on teaching Humanitude™, performed the training to participants.

**Fig. 1:**
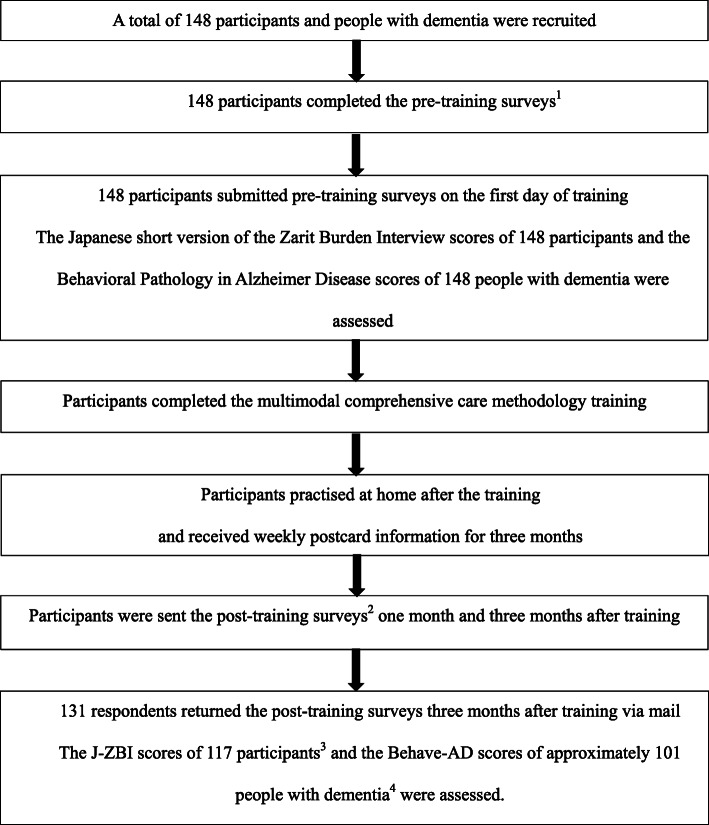
Procedure of the multimodal comprehensive care methodology training programme. 1: Pre-training surveys: Completion of the Japanese short version of the Zarit Burden Interview (J-ZBI) by the participants and the Behavioral Pathology of Alzheimer’s Disease by the participants regarding people with dementia. Completion of the background information of family caregivers, including the caregiver’s age, gender and relationship with the person with dementia, and the background information of the people with dementia, including age, gender, aetiology of dementia, support or care need levels under the public long-term care insurance system in Japan, and the use of any sleep or antipsychotic medications. 2: Post-training surveys: completion of the Japanese short version of the Zarit Burden Interview by the participants and the Behavioral Pathology of Alzheimer’s Disease by the participants with regard to people with dementia 1 and 3 months after training. 3: Fourteen participants were excluded due to missing data. 4: Twenty-one people with dementia were excluded due to missing data, hospital admission, or death

One month and 3 months after the training, post-training surveys were performed to obtain the post-intervention J-ZBI scores of the participants and the post-intervention Behave-AD scores of the people with dementia (Fig. 1). The same family caregivers performed all pre- and post-training surveys.

### Data collection

Participants provided pre-training data directly to researchers on the first day of training, including the pre-intervention J-ZBI scores of the participants, the pre-intervention Behave-AD of the person with dementia and the characteristics data of family caregivers and the people with dementia. One month and 3 months after the training, participants sent post-training data to researchers, including the post-intervention J-ZBI scores and post-intervention Behave-AD scores via mail.

### Instruments

#### J-ZBI

The ZBI is one of the most widely used measures of caregiver burden and assesses the impact of caregiving on caregivers, including physical, mental, social and economic aspects [16]. In this study, the short version of the J-ZBI, which has been linguistically validated, was used [17]. This questionnaire is an 8-item instrument that has been widely used and validated among caregivers. It uses a 5-point Likert scale anchored by “strongly disagree” and “strongly agree” (range: 0–32). The participants completed the J-ZBI. The scale was administered before the training (pre-training), 1 month later, and 3 months later (post-training).

#### Behave-AD

The Behave-AD is the most widely used instrument for the evaluation of dementia-related behavioural changes based on informant interviews [18]. The questionnaire is a 26-item instrument that has been widely used and validated among caregivers and uses a 4-point scale. It addresses delusions, hallucinations, activity disturbances, aggressiveness, diurnal rhythm disturbances, affective disturbances, anxieties and phobias. The people with dementia were assessed using the Japanese version of the Behave-AD, which has been linguistically validated [19]. The assessor of each patient was his or her participating family caregiver; the Behave-AD was completed prior to the study and 1 and 3 months after the intervention.

### Outcome measures

The primary outcome was caregiver burden as measured using the J-ZBI scores from before the training (pre-training) to 3 months later (post-training). The secondary outcome was behavioural changes in people with dementia as measured using the Behave-AD scores between pre-training and post-training.

### Statistical analysis

These analyses were performed using R statistical software (version 3.5.3). The characteristics data of family caregivers and people with dementia were analysed using descriptive statistics. Analytical statistics were used to address the primary and secondary outcomes. The normality of all data was verified by the Shapiro-Wilk test. A Wilcoxon signed rank test was used to test for significant differences between the pre-training and post-training J-ZBI scores and Behave-AD scores. The paired-samples t-test was used to test for significant differences in the categories of the J-ZBI scores. The baseline characteristics of the people with dementia were compared using the Fisher’s exact test for categorical variables and the Mann-Whitney U test for continuous variables. Statistical significance was defined when *P* < 0.05.

## Results

A total of 148 family caregivers were enrolled in the study to complete the multimodal comprehensive care methodology training programme. The family caregivers were an average age of 59 years (SD = 11.7). Most family caregivers were female (81%). Seventy-five caregivers (64%) were children of the people with dementia. Twenty-three caregivers (20%) were a spouse of the person with dementia.

### Comparison of the J-ZBI scores

Among the 148 participants, the post-training response rate was 131 (89%). In the analysis of the J-ZBI scores, 14 participants were excluded due to missing data. A total of 117 (79%) patients were assessed after these adjustments.

The distribution of the participants and the results of the pre-training and the post-training J-ZBI scores are shown in Table 1. The J-ZBI scores showed a statistically significant improvement (from 13.3 to 10.6, *p* < 0.05) from pre-training to post-training. Decreases in scores from pre- to post-training were observed among the participants regardless of age, gender, and spouse-child relationship. In particular, there were significant reductions in the care burden among participants younger than 65 years, participants older than 65 years, female participants, and children of people with dementia.

**Table 1:** Pre-training and post-training results of the Japanese short version of the Zarit Burden Interview scores

		Mean Score (95% CI)			
	***n***	Pre-intervention	1 month	3 months	***P***
Zarit Burden Interview score	117	13.3	10.9	10.6	**< 0.001**^**a**^
Over 65 years old	45	13.3	10.9	10.7	**0.02**^**a**^
Under 65 years old	72	13.2	10.9	10.5	**< 0.001**^**a**^
Male	22	13.4	11.0	10.7	**0.09**^**b**^
Female	95	13.3	10.9	10.6	**< 0.001**^**a**^
Spouse	23	13.5	11.0	10.8	**0.09**^**b**^
Child	75	13.3	10.9	10.6	**< 0.001**^**a**^

The bold numbers are significant *P*-values (*p* < 0.05) before training and three months after training.

^a^ Wilcoxon signed rank test

^b^ Paired-samples t-test

### Comparison of the BEHAVE-AD scores

Among the 148 people with dementia, the post-training response rate was 82% (122 patients). The characteristics of the 148 patients are described in Table 2. In the analysis of the Behave-AD scores, some people were excluded due to the following reasons: non-response (26 patients), missing Behave-AD data (19 patients), hospital admission (1 patient) and death (1 patient). A total of 101 people were assessed after these adjustments. Sixty-eight (67.3%) people were women, 36 (35.6%) were 75–84 years old, and 47 (46.5%) were over 85 years old. The average age was 83 years (SD = 7.9). The most common cause of dementia was Alzheimer’s disease (*n* = 42, 41.6%). The people’s support and care need levels were collected. The public long-term care insurance system in Japan classifies frail older adults according to seven levels (‘support need levels’ 1 and 2 and ‘care need levels’ 1–5, where larger numbers indicate more severe need) using a nationally standardized and validated algorithm. The level is determined according to older adults’ physical and mental care needs [20]. In Japan, older people who were classified as higher than care need level 3 were found to be more likely to be institutionalized than those who were classified as care need level 2 or lower [21]. Forty-six people (45.5%) had care needs levels of 3, 4, and 5. Thirty-seven people (36.6%) had taken any sleep medications or antipsychotic medications.

**Table 2:** Characteristics of the people with dementia

	Total (***n*** = 148)	Data available (***n*** = 101)	Lost to follow up^**a**^ (***n*** = 47)	***P***^**b**^
Age, *n* (%)				
65–74 years	28 (18.9)	18 (17.8)	10 (21.3)	0.06
75–84 years	51 (34.5)	36 (35.6)	15 (31.9)	0.5
≥ 85 years	69 (46.6)	47 (46.5)	22 (46.8)	0.29
Women, *n* (%)	102 (68.9)	68 (67.3)	34 (72.3)	0.57
Aetiology of dementia, *n* (%)				
Alzheimer’s disease	63 (42.6)	42 (41.6)	21 (44.7)	0.73
Lewy body dementia	9 (6.1)	8 (7.9)	1 (2.1)	0.27
Vascular dementia	10 (6.8)	9 (8.9)	1 (2.1)	0.17
Other type of dementia	4 (2.7)	2 (2.0)	2 (4.3)	0.59
Dementia with undetermined aetiology	62 (41.9)	40 (39.6)	22 (44.7)	0.48
Support need level, n (%)				
Level 1	9 (6.1)	7 (6.9)	2 (4.3)	0.71
Level 2	5 (3.4)	2 (2.0)	3 (6.4)	0.32
Care need level, n (%)				
Level 1	40 (27.0)	28 (27.7)	12 (25.5)	0.84
Level 2	21 (14.2)	11 (11.0)	10 (21.3)	0.13
Level 3	33 (22.3)	23 (22.8)	10 (21.3)	1.0
Level 4	16 (10.8)	15 (14.9)	1 (2.1)	0.02
Level 5	9 (6.1)	8 (7.9)	1 (2.1)	0.27
Medication, n (%)	59 (39.9)	37 (36.6)	22 (46.8)	0.28

^a^ Lost to follow up: 26 patients were excluded due to non-response, 19 patients were excluded due to missing Behave-AD data, 1 patient was excluded due to hospital admission and 1 patient was excluded due to death

^b^ The Mann-Whitney U test and Fisher’s exact test were used to compare the available data and the data of those lost to follow up. The threshold for statistical significance was set at *p* < 0.05

The results of the pre-training and post-training Behave-AD scores are shown in Table 3. The Behave-AD scores showed a statistically significant improvement (from 13.6 to 11.8, *p* < 0.05) from pre-training to post-training. There were significant improvements in dementia-related behavioural changes among people who needed high levels of care and people who had taken sleep medications or antipsychotic medications.

**Table 3:** Mean pre-training and post-training Behavioral Pathology in Alzheimer’s Disease scores (*n* = 101)

		Mean Score (95% CI)			
		Pre-intervention	1-month	3-months	***P***^**a**^
Behave-AD	101	13.6	11.3	11.8	**0.005**
Alzheimer’s disease	42	13.7	11.4	11.9	**0.07**
Care need levels ≧3	46	13.6	11.3	11.8	**0.004**
Medicine	37	13.7	11.4	11.9	**0.003**

The bold numbers are significant *P*-values (*p* < 0.05) before training and three months after training

^a^ Wilcoxon signed rank test

## Discussion

The findings of this study show that multimodal comprehensive care communication training for family caregivers decreased the care burden of caring for people with dementia. There were a greater number of communication training studies that examined care burden, psychological distress and challenging behaviour in samples of family caregivers. However, few RCT studies demonstrated a reduction in family caregiver burden [22, 23]. Previous studies did not clearly show whether communication training interventions had a tangible impact on the development of specific communication skills that are consistently translated into practice [24]. Morris et al. [24] noted that one of the reasons for this lack of tangible impact in many controlled studies of interventions for caregivers may be that communication skills training is often one aspect of multi-component training programmes.

Our findings are similar to two previous communication training interventions that were evaluated using RCTs that were particularly effective in reducing caregiver burden [22, 23]. These studies also involved the development of practical skills and active participation by caregivers. Active participation includes practicing skills during training and applying these skills and knowledge at home. However, there were differences between these two intervention studies and our intervention in the use of survey assessment, and our study used J-ZBI for assessments. The two prior interventions were not supported by a clear conceptual basis of communication skills [22, 23]. The proposed methodology in the present study is supported by the Humanitude™ concept. This methodology training provides practical care communication skills based on a neurological theory of dementia and humanist philosophy, and it includes lectures on dementia-related knowledge and care [14]. Interpersonal and family contexts are contributory factors in the development and course of BPSDs [25, 26]. The goal of the proposed methodology is to build a good relationship between caregivers and care receivers while creating a sense of sharing a good time together in the process. Caregivers experience greater fulfilment in their care when they feel that their method of caring for patients is effective.

Our study revealed that there was a reduction in the care burden among family caregivers regardless of age, gender, and spouse-child relationship. A majority of studies on the gender of caregivers have reported a higher burden among females [27]. There is also some evidence that caring for an older adult spouse with a disability or chronic condition increases one’s risk of impaired physical and mental health [28, 29]. Despite this evidence, our present results suggest that this methodology training can be used for diverse family caregivers. The reason is that this methodology is considered not intensive and time-consuming but rather is a widely deliverable and sustainable approach for family caregivers; in addition, the weekly postcards after each monthly 2-h training session provided a reminder of the methodology for family caregivers over 3 months.

This study documented post-training diminution of behavioural and psychological symptoms of people with dementia as well as a reduction of the family caregiver burden. However, to the best of our knowledge, no communication training studies resulted in statistically significant changes in the behavioural and psychological symptoms of people with dementia and the family care burden. Our study showed that the post-training Behave-AD scores of people with dementia who needed high levels of care were lower than their pre-training scores. Considering that behavioural problems and psychological symptoms were found to be the primary factor associated with caregiver burden for caregivers of people with dementia [30], the present results may suggest that the multimodal comprehensive care methodology training decreased the care burden of family caregivers by controlling the neuropsychiatric symptoms of people with dementia. In addition, our study also revealed that the Behave-AD post-training scores of people with dementia who had taken any sleep medications or antipsychotic medications were lower than their pre-training scores. These medications often have been used to reduce psychosis and sleep disturbance. However, pharmacological options have modest to no benefits compared to those of placebo but have serious risks, including mortality in older adults with dementia [31]. Our present results may suggest that the methodology is one of the management options for BPSDs. However, our results about the Behave-AD scores are of serious concern given that 47 patients were lost to follow up among the 148 patients with dementia. Twenty-six patients were excluded due to non-response, and 19 patients were excluded due to missing data. First, approximately half of the people with dementia were over 85 years old, and approximately 25% of people had a care need level of 3 or greater. Therefore, we may assume that people with dementia were institutionalized during the study period. Second, lengthy questionnaires, like the Behave-AD, may have resulted in reduced completion, and missing data [32]. We have no data to support these explanations and can only speculate. For patient characteristics, the number of people with dementia in care need level 4 that were included in the post data was higher than the number of people in care need level 4 who were lost to follow up. On the other hand, patient characteristics such as age, gender, aetiology of dementia, support need level 1 or 2, and care need level 1, 2, 3 or 5 showed no significant differences between the included patients and those lost to follow up. The results regarding the patient characteristics are not sufficient to reveal the similarity between the two groups. Therefore, the Behave-AD post-training data likely showed an overestimated intervention effect. Further studies are needed with a control group to reveal the relevance of the intervention.

Several limitations should be discussed. First, this study was an intervention trial with a quasi-experimental design, not a randomized controlled trial. Therefore, it is possible that confounding factors influenced the association between the training and the J-ZBI and Behave-AD scores. Further randomized controlled studies with larger sample sizes are required. Second, the sample size of this study was small. Third, the outcome assessment time frame in this study might be a weakness with respect to the assessment of care burden. Our study showed that the Behave-AD scores 3 months after training were slightly higher than those 1 month after training. While family caregiver burden may have been significantly reduced immediately after training, the study provides limited insights about the long-term efficacy of the training.

## Conclusions

This study was conducted to assess the effectiveness of a multimodal comprehensive care methodology training to reduce the care burden of family caregivers. The multimodal comprehensive care methodology training was associated with a reduction of the care burden of family caregivers. Because this was a single-arm, pre-post study, the findings suggest that randomized controlled trials with larger sample sizes are needed.

## Data Availability

The datasets used during the current study are available from the National Hospital Organization Tokyo Medical Center, but restrictions apply regarding the availability of these data and they are not publicly available. However, the data are available from the corresponding author upon reasonable request and with permission from the National Hospital Organization Tokyo Medical Center.

## Abbreviations

J-ZBI: Japanese short version of the Zarit Burden Interview
Behave-AD: Behavioral Pathology in Alzheimer’s Disease
BPSDs: Behavioural and Psychological Symptoms of Dementia
